# Evaluation of the malaria rapid diagnostic test SDFK90: detection of both PfHRP2 and Pf-pLDH

**DOI:** 10.1186/1475-2875-11-359

**Published:** 2012-10-29

**Authors:** Marloes Heutmekers, Philippe Gillet, Lieselotte Cnops, Emmanuel Bottieau, Marjan Van Esbroeck, Jessica Maltha, Jan Jacobs

**Affiliations:** 1 Department of Clinical Sciences, Institute of Tropical Medicine, Antwerp, Belgium

**Keywords:** Malaria, Plasmodium falciparum, Diagnosis, Rapid diagnostic test, Histidine-rich protein, *Plasmodium falciparum*-specific *Plasmodium* lactate dehydrogenase, Evaluation

## Abstract

**Background:**

Rapid diagnosis of *Plasmodium falciparum* infections is important because of the potentially fatal complications. SDFK90 is a recently marketed malaria rapid diagnostic test (RDT) targeting both histidine-rich protein 2 (PfHRP2) and *P*. *falciparum*-specific *Plasmodium* lactate dehydrogenase (Pf-pLDH). The present study evaluated its diagnostic accuracy.

**Methods:**

SDFK90 was tested against a panel of stored whole blood samples (n= 591) obtained from international travellers suspected of malaria, including the four human *Plasmodium* species and *Plasmodium* negative samples. Microscopy was used as a reference method, corrected by PCR for species diagnosis. In addition, SDFK90 was challenged against 59 *P*. *falciparum* samples with parasite density ≥4% to assess the prozone effect (no or weak visible line on initial testing and a higher intensity upon 10-fold dilution).

**Results:**

Overall sensitivity for the detection of *P*. *falciparum* was 98.5% and reached 99.3% at parasite densities >100/μl. There were significantly more PfHRP2 lines visible compared to Pf-pLDH (97.3% *vs* 86.9%), which was mainly absent at parasite densities <100/μl. Specificity of SDFK90 was 98.8%. No lot-to-lot variability was observed (*p* = 1.00) and test results were reproducible. A prozone effect was seen for the PfHRP2 line in 14/59 (23.7%) *P*. *falciparum* samples tested, but not for the Pf-pLDH line. Few minor shortcomings were observed in the kit’s packaging and information insert.

**Conclusions:**

SDFK90 performed excellent for *P*. *falciparum* diagnosis. The combination of PfHRP2 and Pf-pLDH ensures a low detection threshold and counters potential problems of PfHRP2 detection such as gene deletions and the prozone effect.

## Background

*Plasmodium falciparum* is the most dangerous malaria species in both endemic and non-endemic settings as it can easily lead to severe complications and death. Therefore rapid diagnosis and treatment are crucial to reduce morbidity and mortality [1]. Although microscopy is the cornerstone of malaria diagnosis, it requires special equipment and expertise, which is often unavailable in both endemic and non-endemic settings. Malaria rapid diagnostic tests (RDTs) are hand-held cassettes containing a nitrocellulose membrane on which detection of *Plasmodium* antigens become visible as blue or cherry-red lines. Two detection antigens exist for *P*. *falciparum* diagnosis: histidine-rich protein-2 (PfHRP2) and *P*. *falciparum*-specific *Plasmodium* lactate dehydrogenase (Pf-pLDH). PfHRP2 is the most frequently used detection antigen and it is known to have a lower detection limit compared to Pf-pLDH [2]. However, Pf-pLDH also has advantages over PfHRP2: it is not affected by the prozone effect [3,4] nor by *pfhrp2* gene deletions [5]. Therefore, a RDT product detecting both PfHRP2 and Pf-pLDH could be of particular interest as the disadvantages of one detection antigen may be countered by the other. The recently marketed RDT product SD Malaria Antigen P.f 05FK90-02-0 (HRP2/pLDH), Standard Diagnostics, Inc., Kyonggi-do, Korea, (hereafter referred to as SDFK90), is a three-band RDT consisting of a control line, a test line with antibodies directed to PfHRP2 and a different test line with antibodies directed to Pf-pLDH. The present study evaluated the diagnostic accuracy of SDFK90 in a non-endemic setting.

## Methods

### Study design

The SDFK90 was evaluated using a panel of stored blood samples in a non-endemic reference laboratory. All samples were obtained from international travellers suspected of malaria. The reference method was microscopy corrected by polymerase chain reaction (PCR) for *Plasmodium* detection and species identification. Microscopy was used to determine parasite densities and stages. The study design was in compliance with the STARD guidelines for presentation of diagnostic studies [6].

### Patients and materials

The evaluation panel was selected from EDTA anti-coagulated whole blood samples. The majority of samples were obtained from patients presenting at the outpatient clinic of the Institute of Tropical Medicine (ITM) in Antwerp, Belgium. An additional part was submitted to ITM by other Belgian laboratories for confirmation in the scope of the national reference laboratory for the diagnosis of malaria. The samples were obtained from international travellers suspected of malaria. Samples were obtained between February 1996 and May 2011. Samples collected at ITM were kept at room temperature, below 25°C, for a maximum of eight hours before being stored at −70°C. Samples acquired from other Belgian laboratories had been exposed to ambient temperatures for the period of shipment which was generally less than 24 hours with a maximum of 48 hours. The selected panel comprised the four human *Plasmodium* species at different parasite densities, as well as *Plasmodium* negative samples confirmed by microscopy, PCR and RDTs used in standard diagnostic work-up. The selected panel also included five samples that showed a prozone effect in a previous ITM study [3]. Samples with pure gametocytaemia were included among the *P*. *falciparum* species. Mixed infections were not considered.

Additionally, samples obtained from patients presenting at the Provincial Hospital of Tete (Mozambique) which had shown a prozone effect in a previous ITM study [4] were used to assess the prozone effect: samples with high parasite densities (≥4%) having no visible, faint or weak test lines and a higher line intensity when tested with a 10-fold dilution [3]. The samples were stored at −70°C for 15–21 months.

### Reference method

Malaria diagnosis at ITM is accredited to the requirements of ISO 15189:2007. An expert microscopist assessed all samples for the presence of *Plasmodium* parasites. Species identification and parasite density were performed according to the WHO standards for microscopy. The only exception was that Giemsa staining was done with pH 8.0 [7,8]. Thick and thin blood films were prepared and examined by light microscopy. A minimum of 200 fields were examined before a blood film was reported negative. The parasite density was obtained by counting the asexual parasites against 200 or 500 white blood cells (WBC) in thick blood films and using the WBC count or, when not available, the standard 8,000 WBC/μl, for the conversion to parasites/μl [7,8]. Four-primer real-time PCR was performed on all samples [9]. The result of microscopy corrected by PCR was considered for species identification. PCR results were used for final species identification; microscopy was used for stage determination (especially gametocytes for *P*. *falciparum*) and determination of parasite density.

### Test platforms

SDFK90 consists of a control line and two test lines: Pf-pLDH and PfHRP2. The test is considered positive for *P*. *falciparum* when the control line is present and one or both test lines. In case of absence of the control line the test was considered invalid and repeated. Kits from two different lot numbers were used: MFRDT1001 (n = 234) and MFRDT1002 (n = 416). RDTs had been stored between 18°C and 24°C.

### Test procedures

Tests were carried out in time-controlled batches and in compliance with the instructions of the manufacturer, except for replacement of the included plastic transfer devices by a transfer pipette (Finnpipette, Helsinki, Finland). Readings were carried out at daylight assisted by a standard light source. Readings were subsequently carried out by three trained observers the first two of whom performed the RDTs. All three observers scored test results within the recommended reading time (15–30 minutes). Photographs of the batches were taken within 15–30 minutes. The observers were blinded to microscopy, PCR and each other’s results.

A scoring system was used to categorize line intensities: negative, faint, weak, medium and strong [10]. Test results were based on consensus, *i*.*e*. an identical score by at least two out of three readers. In case of discordances and absence of consensus, the photographs were reviewed to conclude.

### Data management and statistical analysis

Data was recorded on register forms and entered in a Microsoft Excel database (Microsoft Corporation, Redmond, Washington, USA). End points were sensitivity, specificity, inter-observer agreement and reproducibility. Sensitivity and specificity were calculated with 95% confidence interval (CI). Proportions were assessed for statistical significance using the two-tailed Fisher’s exact test and the McNemar test, for unpaired and paired panels respectively. A *p*-value < 0.05 was considered significant.

Inter-observer agreement for both positive and negative readings as well as for line intensity was expressed by the percentage of overall agreement and by kappa values for each pair of observers. A Kappa value of 0.6-0.8 was considered good, > 0.8 was considered excellent [11]. Test reproducibility was evaluated by testing 15 samples representing all species at varying parasite densities on six occasions.

### Additional analysis

PfHRP2 antigen qualitative ELISA (SD Malaria Antigen P.f ELISA, Standard Diagnostics, Inc., Kyonggi-do, Korea) was performed in case of a visible PfHRP2 line among *Plasmodium* negative samples or non-*falciparum* infections, to determine the presence of circulation PfHRP2 antigen. All false negative *P*. *falciparum* samples and non-*falciparum* samples that generated a PfHRP2 and/or Pf-pLDH line were retested with the SDFK90.

### Assessment of the prozone effect

PfHRP2 RDTs may be affected by the prozone effect, a false-negative or false-low result due to an excess of antigens [3]. To assess the susceptibility of SDFK90 for the prozone effect, samples with high parasitaemia (n = 59, parasite density ≥ 4%) that showed a prozone effect in previous ITM studies were assessed with SDFK90 side-to-side with two PfHRP2-detecting RDTs, Paracheck Pf (PfHRP2, Orchid Biomedical Systems, Goa, India, lot number 31797) and ICT Pf (PfHRP2, ICT Diagnostics, Cape Town, South Africa, lot numbers 50045 and 32784). Whole blood samples were tested as well as a 10x dilution with NaCl 0.9%. RDTs were performed as described above, except that the reading was done by two, instead of three, observers. A prozone effect was defined as a sample with a negative, faint or weak test line when tested undiluted, and a visible test line of higher intensity at 10-fold dilution, as observed by two blinded observers [4].

### Package, labelling and instructions for use

Checklist for assessing quality of packaging, labelling and instructions were applied [12]. The Flesh Kincaid Grade Level was used to score the readability of the manufacturer’s instructions: it indicates the number of years of education that is needed to understand the text, based on measures of word and sentences length [12]. In addition, letter type (open *versus* closed), font size, and inter-line spacing were assessed as previously described [12].

### Ethical review

The study was approved by the Institutional Review Board of ITM and by the Ethical Committee of Antwerp University, Belgium.

## Results

### Sample collection

The collection consisted of 591 samples obtained in 591 patients with a median age of 36 years (range five months to 85 years), and a male to female ratio of 1.7:1. Eight children (1.4%) under the age of five were included. In 492 (83.2%) patients the travel history was known, 86.6% (426/492) of them had recently returned from Sub-Saharan Africa and 9.1% (45/492) from Asia. The samples included 336 *P*. *falciparum*, 72 *Plasmodium vivax*, 71 *Plasmodium ovale* and 16 *Plasmodium malariae* samples. Microscopic species identification was corrected by PCR in seven out of the 495 positive samples (1.4%). This correction only comprised *P*. *vivax* - *P*. *ovale* mismatches: in the final collection, two out of 72 (2.8%) *P*. *vivax* samples and five out of 71 (7.0%) *P*. *ovale* samples had been identified respectively as *P*. *ovale* and *P*. *vivax* by microscopy. Among the *P*. *falciparum* samples, there were 18 with pure gametocytaemia as identified by microscopy. The median parasite density of the remaining 318 *P*. *falciparum* samples was 4,876.5/μl (range 6–1,750,000/μl). The median (range) parasite densities for *P*. *vivax*, *P*. *ovale* and *P*. *malariae* were 1,834/μl (15–32,000/μl), 609/μl (10–10,000/μl), and 473/μl (0.1–6,096/μl) respectively. In addition, 96 microscopic and PCR malaria negative samples of symptomatic travellers were included in the panel.

The sample collection for assessment of the prozone effect consisted of 59 *P*. *falciparum* samples mainly obtained in malaria suspected patients in Mozambique (n=54) and at ITM (n=5). The parasite density ranged from 200,000–1,750,000 parasite/μl (median 460,000). The median age of these patients was three years (range three months to 73 years), 69.5% (41/59) was <5 years of age. The male to female ratio was 0.97:1.

### Test characteristics

No invalid test results were obtained. The majority of the *P*. *falciparum* samples (288/336, 85.7%) showed both PfHRP2 and Pf-pLDH test lines (Table 1). Of the five *P*. *falciparum* samples not detected by SDFK90, two had pure gametocytaemia, the other three had parasite densities of 83/μl, 703/μl and 2,043/μl.

**Table 1:** Test results of SDFK90 for all samples (n = 591), except for samples assessed for the prozone effect

	**PfHRP2 line positive**		**PfHRP2 line negative**	
	**Pf-pLDH line positive**	**Pf-pLDH line negative**	**Pf-pLDH line positive**	**Pf-pLDH line negative**
*P*. *falciparum* (n = 336)	288	39^*^	4^†^	5^‡^
*P*. *vivax* (n = 72)	1^§^	2^§^		69
*P*. *ovale* (n = 71)				71
*P*. *malariae* (n = 16)				16
Negative (n = 96)				96

^*^ five samples with pure gametocytaemia, other samples parasite density: 6/μl–2,622/μl (median 71/μl).

^†^ two samples with pure gametocytaemia, other samples parasite density: 238/μl, 1,123/μl.

^‡^ two pure gametocytaemia, other samples parasite density 83/μl, 703/μl, 2,043/μl.

^§^ Species mismatch.

Overall sensitivity for the detection of *P*. *falciparum* for the two test lines combined was 98.5% and increased to 99.3% at parasite densities >100/μl (Table 2). Overall sensitivity for the PfHRP2 test line (97.3%) was significantly higher compared to that of the Pf-pLDH test line (86.9%, *p* = 0.0001). When the samples with pure gametocytaemia were subtracted it was clear that this difference was mainly caused by differences in sensitivity at parasite densities below 100/μl (Table 3). There was no difference (*p* = 1.00) in diagnostic sensitivity between the two lots tested.

**Table 2:** **Sensitivity and specificity of SDFK90 for the detection of *Plasmodium falciparum***

**Microscopy corrected by PCR**	**Number**	**Identified as *P *. *falciparum *by SDFK90**	**% Sensitivity (95% CI)**	**% Specificity (95% CI)**
*All P*. *falciparum* samples	336	331	98.5 (96.6-99.5)	
Pure gametocytaemia	18	16	88.9 (65.3-98.6)	
Asexual parasite density 1-100/μl	36	35	97.2 (85.5-99.9)	
Asexual parasite density 101-200/μl	19	19	100 (82.4-100)	
Asexual parasite density 201-1,000/μl	44	43	97.7 (88.0-99.9)	
Asexual parasite density >1,000/μl	219	218	99.5 (97.5-100)	
Asexual parasite density >100/μl	282	280	99.3 (97.2-99.9)	
Excluding pure gametocytaemia	318	315	99.1 (97.3-99.8)	
All other species and no parasites detected	255	3		98.8 (96.6-99.8)
No parasites detected	96	0		
*P*. *vivax*	72	3		
*P*. *ovale*	71	0		
*P*. *malariae*	16	0		

**Table 3:** **Sensitivities of both test lines separately for the detection of *Plasmodium falciparum***

**Microscopy corrected by PCR**	**Number**	**% Sensitivity PfHRP2 line (95% CI)**	**% Sensitivity Pf-pLDH line, (95% CI)**	**McNemar (p-value)**
All *P*. *falciparum* samples	336	97.3 (95.0-98.8)	86.9 (82.8-90.3)	0.0001
Pure gametocytaemia	18	77.8 (52.4-93.6)	61.1 (35.8-82.7)	0.4497
Asexual parasite density 1-100/μl	36	97.2 (85.5-99.9)	30.6 (16.4-48.1)	0.0001
Asexual parasite density 101-200/μl	19	100 (82.4-100)	73.7 (48.8-90.9)	0.0736
Asexual parasite density 201-1,000/μl	44	95.5 (84.5-99.4)	88.6 (75.4-96.2)	0.3711
Asexual parasite density >1,000/μl	219	99.1 (96.7-99.9)	99.1 (96.7-99.9)	0.4795
Asexual parasite density >100/μl	282	98.6 (96.4-99.6)	95.7 (92.7-97.8)	0.0433
Excluding pure gametocytaemia	318	98.4 (96.4-99.5)	99.1 (97.3-99.8)	0.4658

Three out of the 72 *P*. *vivax* samples (parasite densities of 568/μl, 916/μl and 3,875/μl) showed a visible PfHRP2 test line and one of them showed an additional Pf-pLDH line. These three samples showed also strong positive results in the PfHRP2 qualitative antigen ELISA. None of the *Plasmodium* negative, *P*. *ovale* and *P*. *malariae* samples showed a visible test line (Tables 1 and 2).

### Intensity of test lines

Among *P*. *falciparum* samples, the majority (283/327, 86.5%) of visible PfHRP2 test lines had medium or strong line intensities. For the Pf-pLDH line, only 45.5% (133/292) of test lines had medium or strong line intensities (*p* = 0.0001), all but two occurred among samples with parasite densities higher than 1,000/μl. In addition, 3.7% of the correctly identified PfHRP2 test lines had faint line intensity, whereas this was 17.8% for the Pf-pLDH line.

### Inter-observer agreement and reproducibility

For both PfHRP2 and Pf-pLDH test lines overall agreement (≥ 98.8%) and kappa values between pairs of observers (≥ 0.97) were excellent for positive and negative readings. For line intensity readings kappa values were good and excellent (0.75–0.92). Consensus in line intensity reading was obtained for all cases, except for three samples and only for the Pf-pLDH line with as respective results for the three observers: faint, negative and weak). After review of the photographs, the faint test line intensity was used as consensus. Test results were reproducible and all discordances in line intensity occurred within one category of difference.

Upon retesting of discordant RDT results, the five false negative *P*. *falciparum* samples as well as the three *P*. *vivax* samples with PfHRP2 and/or Pf-pLDH lines scored identical to initial testing.

### Assessment of the prozone effect

A prozone effect was seen in the PfHRP2 test line of 14/59 (23.7%) samples tested by SDFK90; all 14 samples generated weak test lines whereas they showed strong line intensities upon 10-fold dilution. The prozone effect was not observed for the Pf-pLDH test line. Paracheck and ICT Malaria Pf showed a prozone effect in 15/58 (25.9%) and 31/58 (53.4%) of samples respectively.

### Package, labelling and instructions for use

The following shortcoming were noted: the SDFK90 kit’s box and labels were not humidity resistant and the names as displayed on information inserts, boxes, the device blisters and buffer vials showed slight differences. The plastic housing of the cassette however was clearly labelled and specified target antigens by their names. The instructions for use did not mention: (i) do not store the test in a freezer, (ii) check the saturation of silica gel, (iii) write the patient’s identification on the cassette; and, (iv) use only the buffer vial provided in the kit. Readability expressed as Flesh Kincaid Grade Level was 11.02, an open letter type was used and font size was 7 with an interline spacing of 1.

## Discussion

The present study assessed the performance of SDFK90 for malaria diagnosis, a RDT detecting both PfHRP2 and Pf-pLDH, using stored samples obtained in international travellers suspected of malaria. Overall sensitivity for *P*. *falciparum* was 98.5% and reached 99.3% at parasite densities above 100/μl. Specificity for non-*falciparum* and *Plasmodium* negative samples was 98.8%.

A number of limitations need to be considered. Due to the use of stored samples, clinical information potentially explaining false positive or false negative results, such as the visible PfHRP2 line among three *P*. *vivax* samples, was not available. Furthermore, because of the use of stored (lysed) blood samples, a calibrated pipette was used instead of the RDT kit’s original transfer device (“loop”). This could bypass possible errors in transfer volume, which may occur when performing the test in a field setting. Finally, the application of stringent interpretation criteria unfavourably influenced test outcomes: *P*. *falciparum* samples with pure gametocytaemia were included among the positive samples. Although meaningful in the scope of travel medicine [13], it added to the false-negative results and decreased the sensitivity of SDFK90.

SDFK90 was previously evaluated in the third evaluation round of the World Health Organization (WHO) and the Foundation for Innovative New Diagnostics (FIND) [14], in which detection of *P*. *falciparum* and *P*. *vivax* was assessed using diluted samples at fixed parasite densities. The detection rate for *P*. *falciparum* was 87.9% and 100% at low (200/μl) and high (2,000/μl or 5,000/μl) parasite densities respectively. In the present study, even at parasite densities <200/μl sensitivity already reached 97.2%.

PfHRP2 detection has advantages over the use of Pf-pLDH antigen detection. That is, PfHRP2-detecting RDTs are generally reported to be more heat resistant, although some Pf-pLDH-detecting RDTs do have extended temperature stabilities [11,14]. The heat stability testing of the WHO/FIND study confirmed resistance of the SDFK90 to temperatures up to 45°C [11,14-16]. Moreover, PfHRP2-detecting RDTs are generally reported to have lower sensitivity for *P*. *falciparum* diagnosis, especially at parasite densities ≤100/μl [2,11,17-19].

On the other hand, detection of the Pf-pLDH antigen also has some advantages over PfHRP2 detection [11]. Unlike the PfHRP2 antigen, Pf-pLDH is not susceptible to the prozone effect, *i. e.*, a missed or delayed diagnosis at high parasite densities, as was confirmed in the present study. Although rare in travel medicine, the consequences of prozone can lead to serious complications and the risks increase when laboratories rely on RDTs alone for diagnosis [3,20]. The detection of both Pf-pLDH and PfHRP2 will offer a back-up in case of a prozone effect. In addition, it will offer a back-up in sam-ples lacking the *pfhrp2* gene [5]: 25.7%–41.0% of *P*. *falciparum* samples in the Peruvian Amazon lack the *pfhrp2* gene which encodes PfHRP2 [5,21]. All PfHRP2-detecting RDTs evaluated by Maltha *et al*. failed to correctly diagnose these samples, whereas SDFK90, like the Pf-pLDH-detecting RDTs, correctly identified the *P*. *falciparum* infections because of the presence of the Pf-pLDH test line [5].

PfHRP2 is produced by asexual parasites and young gametocytes. It is expressed on the red blood cell membrane and readily diffuses in the plasma. Due to its slow clearance, PfHRP2 antigen persists in the bloodstream for up to several weeks after successful treatment of the infection [22-24]. Pf-pLDH is an enzyme of the glycolytic pathway produced by asexual stages and gametocytes and its presence depends on living parasites. Pf-pLDH quickly disappears from the blood when parasites have been cleared [2,25].

Although the Pf-pLDH antigen is rapidly cleared from the blood once treatment is initiated [22-24], its use for treatment follow-up has been abandoned because of the persistence of gametocytes for up to two weeks after the start of the treatment [26,27]. On the other hand, artemisinin-based combination therapy results in low post-treatment gametocytaemia with Pf-pLDH antigen levels below the RDT detection threshold [28]. While the use of Pf-pLDH to follow-up treatment and detection of therapy resistance is still under debate and awaiting further study, it can be argued that the combination of PfHRP2 and Pf-pLDH in the SDFK90 could offer an opportunity for treatment follow-up.

As expected, the specificity of the Pf-pLDH line was higher compared to the PfHRP2 line [17]. This can be explained by the persistence of PfHRP2 antigen for up to several weeks after successful treatment. Due to the retrospective design of this study, clinical information explaining the reactions of the PfHRP2 line with three *P*. *vivax* samples was not available, but the identical positive result in PfHRP2 ELISA suggests PfHRP2 persistence after a previous *P*. *falciparum* infection.

Line intensities of Pf-pLDH were lower than those of PfHRP2, irrespective of parasite densities. In previous studies, the ITM team consistently found lower line intensities in pLDH test lines [10,11,29-33]. In particular weak and faint line intensities are of concern, as they tend to be regarded as negative [34]. The observed shortcomings in instructions for the use and labelling of the kit’s package and contents were in line with observations made for other RDT kits [12] and can easily be corrected.

## Conclusion

SDFK90 performed excellently for the diagnosis of *P*. *falciparum*. The detection of both PfHRP2 and Pf-pLDH ensures a low detection threshold and minimizes potential problems of PfHRP2 detection such as gene deletions and the prozone effect. Further evaluation of SDFK90 in an endemic setting is needed to determine predictive values and its possible use for treatment follow-up.

## Abbreviations

CI: Confidence interval; EDTA: Ethylene diamine tetra-acetic acid; FIND: Foundation for innovative new diagnostics; ELISA: Enzyme linked immuno sorbent assay; ISO: International organization for standardization; ITM: Institute of tropical medicine; P: Plasmodium; Pan-pLDH: pan *Plasmodium* lactate dehydrogenase; PCR: Polymerase chain reaction; PfHRP2: *P*. *falciparum* Histidine-rich protein 2; Pf-pLDH: *Plasmodium falciparum*-specific *Plasmodium* lactate dehydrogenase; pLDH: *Plasmodium* lactate dehydrogenase; RDT(s): Rapid diagnostic test(s); STARD: Standards for the reporting of diagnostic accuracy studies; WHO: World health organization.

## Competing interests

The authors declare that they have no competing interests.

## Authors' contributions

PG and JJ designed the study protocol. MVE, JJ and EB organized prospective sample collection. MH and PG carried out the RDT test evaluations, LC performed PCR analysis. MH, PG, JM and JJ analysed and interpreted the results. MH, PG, JM and JJ drafted the manuscript. All authors critically reviewed the manuscript and approved the final manuscript.
